# Ecosystem restoration as a boundary object, demonstrated in a large-scale landscape restoration project in the Dovre Mountains, Norway

**DOI:** 10.1007/s13280-021-01582-2

**Published:** 2021-06-17

**Authors:** Jørund Aasetre, Dagmar Hagen, Kristine Bye

**Affiliations:** 1 Department of Geography, Norwegian University of Science and Technology, 7491 Trondheim, Norway; 2 Norwegian Institute for Nature Research, Torgard, P.O. Box 5685, 7485 Trondheim, Norway; 3 The Directorate of Mining with the Commissioner of Mines at Svalbard, Ladebekken 50, NO-7066, Trondheim, Norway

**Keywords:** Dovre Mountain, Ecosystem restoration, Mutual understanding, Nature management, Restoration conflicts, Stakeholders

## Abstract

Coordinating and implementing ecosystem restoration projects can be challenging when the professions involved have differing perceptions of ecological restoration and implementation in practice. To overcome these barriers in complex restoration projects, we suggest analysing ecosystem restoration as a boundary object, a concept drawn from the field of science and technology studies. We use a large scale restoration project in the Dovre Mountains of Norway to demonstrate the validity of using the boundary object concept in this context. The restoration involves a former military training area where the goal of the project was to protect and restore the environment and allow for civilian use. We examine how the different professions developed sufficient mutual understanding to make the project work. In particular, we explore the extent to which the perceptions of different professions overlap, the diversity of the perceptions in the project and how this might influence the outcome of the restoration. The boundary object concept offers potential to help improve restoration quality and reduce conflicts.

## Introduction

Over the last decade, ecosystem restoration has been acknowledged in global and regional policy, and in science as a crucial activity to reverse the amount of degraded land and to ensure the continued provision of ecosystem services (e.g., Benayas et al. 2009; CBD 2010; Comín 2010; Bullock et al. 2011). The Intergovernmental Panel on Climate Change (IPCC 2019) and the Intergovernmental Science-Policy Platform on Biodiversity and Ecosystem Services (IPBES 2019) have stated that the restoration of natural areas and species is needed to protect the climate and biodiversity and to achieve the UN Sustainable Development Goals, in particular SDG 13 (climate action), SDG 14 (life below water) and SDG 15 (life on land) (UN 2019). The UN Assembly has declared 2021–2030 as the decade of restoration, aiming for a “massive upscaling of restoration” (UNEP 2020).

This increased attention has contributed to a large increase in active restoration projects, and to a shift in restoration goals from focusing solely on biodiversity, to a broader approach of securing the supply of ecosystem services (e.g., Lindenmayer et al. 2012). The expected future upscaling of restoration will likely cause controversies from land-use pressure and conflicting priorities between different interests, because restored areas will replace other types of land use (Tolvanen & Aronson 2016). Ecosystem restoration is not an obvious activity in terms of objectives, targets, or tools (Jørgensen 2015). The different actors involved, value considerations, variation in spatial and temporal scales, methods and goals of these projects can be highly diverse. In consideration of the UN goal of a “massive upscaling of restoration” (UNEP 2020) these activities need to be approached with new concepts and theories to understand conflicts and find multi-use solutions.

Ecological restoration involves activities and processes that assist the recovery of degraded, damaged or destroyed ecosystems to improve biodiversity, human health, and ecosystem services (SER 2004; Gann et al. 2019). The interaction between different professions in these projects is crucial, and within specific restoration project people with different backgrounds and skills are bound together in a common task. When different professions are involved, concepts and contents may be perceived differently by different groups (Dephner and Haase 2018, Kaltenborn and Bjerke 2002).

This study introduces the concept of boundary objects (Star and Griesemer 1989; Star 2010) to analyse the interface between science and policy as demonstrated in a large-scale landscape restoration project. We hypothesize that ecological restoration as a management enterprise may have a communicative and coordinative function between actors with different positions and interests, and in so doing, function as a boundary object.

In 1999, the Norwegian Parliament decided to phase out one of the largest military training areas in the country to restore the area for civilian use and turn it into a national park (Norwegian Defence Estate Agency 2020). The project was ground-breaking due to its size, the high level of ambition (i.e., future nature protection), and was undertaken in a remote area with a harsh climate and a history of military use, which required a special emphasis on safety for future users. In addition, Norway has a limited history of undertaking restorations, and both the public and the authorities have limited awareness of and experience with the concept and goals of restoration (Hagen et al. 2013).

The boundary object approach to restoration offers a new perspective on understanding alliances and the positions of involved parties, and a way to analyse how actors with different backgrounds, interests and perceptions can be coordinated and work together. This paper demonstrates how the theory of boundary objects can contribute to the understanding and explanation of processes, success and failures in restoration projects. The use of a specific restoration case project allows us to identify a conceptual core, or storyline (Mäntysalo et al. 2020), with communicative and coordinating capacity to be seen as a boundary object. We discuss how the analytical use of the boundary object concept can contribute to improve the quality of restoration projects in general and reduce conflicts and misapprehensions.

## Theory

### Boundary objects

The concept of boundary objects has been applied to scientific work in complex settings (Star and Griesemer 1989; Star 2010). Bowker and Star (2000, p. 297) describe boundary objects as “those objects that both inhabit several communities of practice and satisfy the informational requirements of each of them”. In an environmental context the concept has been used to articulate uncertainties and conflicts at the science–policy interface (van der Sluijs 2006; Jørstad and Skogen 2010; Bye-Larsen 2012), and in the use of ecological indicators to evaluate the effectiveness of policy and management actions (Turnhout 2009).

When different professions and interest groups are involved in effort such as restoration projects, the different players may perceive both concepts and contents ae differently. If we perceive ecosystem restoration as a boundary object, the different positions involved in a restoration project, such as restoration ecologists, technical engineers, contract entrepreneurs, bureaucrats and stakeholders in the local communities, need to be coordinated. Landscape architects, who are often involved in restoration projects involving recreation or urban habitats, did not play a role in our case project, as the main goal here was the restoration of wilderness and ecosystem functions. As a boundary object, ecosystem restoration planning and implementation should be “both plastic enough to adapt to local needs and constraints of the several parties employing them, yet robust enough to maintain a common identity across sites. … They have different meanings in different social worlds but their structure is common enough to more than one world to make them recognizable, a means of translation” (Star and Griesemer 1989, p. 393). This description can be seen as a working definition of the concept. Within this frame, ecosystem restoration fits with how other concepts, such as “stewardship” (Enqvist et al. 2018), or “ecosystem services” (Steger et al. 2018) have employed a boundary object perspective. Restoration ecologists might think their training as scientists gives them the “objective knowledge” best suited to decide what ecosystem restoration should entail. However, in an on-the-ground restoration project, this position may be contested and challenged by people representing other social perspectives, such as local user groups, construction companies or government bureaucrats.

The boundary object approach to restoration can offer a new perspective for understanding situations where actions have to be coordinated between actors and stakeholders with conflicting goals and values. Mäntysalo et al. (2020) described storylines in planning processes as boundary objects that coordinate discourses and actions between involved actors. As an example, they described how the concept of a “growth axe” was employed in developing the centre of the city of Aalborg. This was an idea that functioned as a catalyst and offered the ability to coordinate discourses and actions regarding how development in that city should proceed. Ecosystem restoration could play a similar role as a coordinating boundary object in a project, depending on the communicative and coordinating “power” of ecosystem restoration to enable this. This article examines the restoration project in the Dovre Mountains in this context as a way to elucidate the analytical power of this concept.

### Ecosystem restoration-different Perspectives

Ecological restoration was founded on an assumption of the recovery for an area’s earlier state and improve the function of degraded ecosystems, with a focus on conserving biodiversity (Jordan et al. 1987). Recently, this focus has been expanded to include anthropogenic impacts, to mitigate specific societal challenges, and to secure the delivery of ecosystem services, including carbon sequestration (Bullock et al. 2011; Elmqvist et al. 2015). Ecological theory and concepts are traditionally the primary design criterions for ecological restoration (Palmer et al. 2005; Young et al. 2005). However, the social-ecological system (SES) sets the context for restoration activities (Aradóttir and Hagen 2013; Baker and Eckerberg 2013; Clewell and Aronson 2007).

The act of restoring an ecosystem involves conflicting goals regarding conceptual dimensions, such as untouched nature or wilderness (Cronon 1996; Arts et al. 2012) versus cultural landscapes (Jones 2003), the debate over restoring an area to its original state versus a normative “desired state” (Hagen et al. 2002) and participation (Arnstein 1969; Cornwall 2008) versus more expert-oriented rational planning (Banfield 1959). These theoretical perspectives offer analytical tools in assessing how different people perceive the goal of specific restoration projects and contribute to understanding of how people in different positions perceive ecosystem restoration in general, and in specific restoration projects, in as much as scientists, bureaucrats, and the public might have different views of what the end results of restoration should be (Casagrande 1997).

## Methods

### Case area and study design

**Fig. 1:**
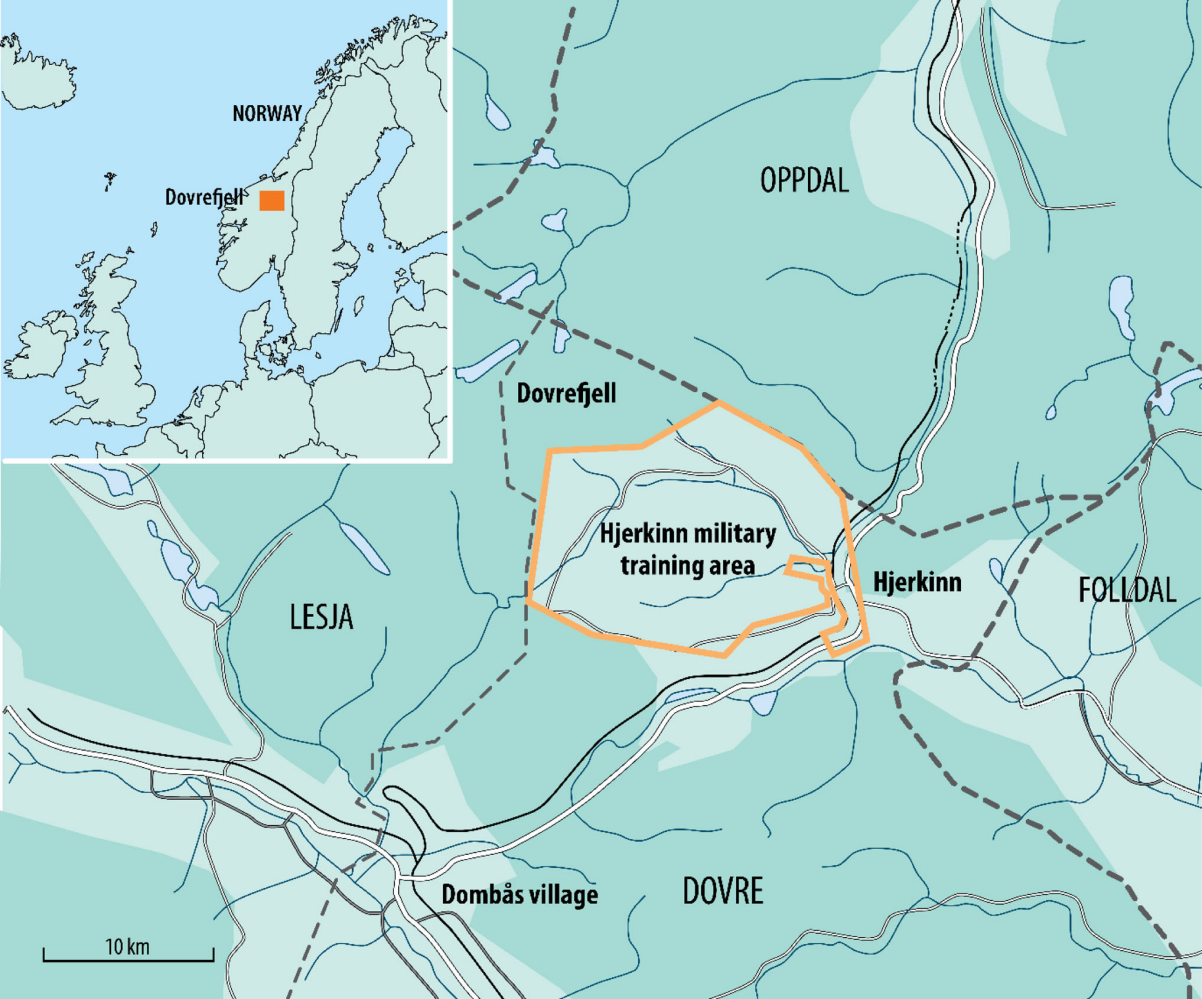
The former Hjerkinn military training area in Dovrefjell (Dovre Mountains), in the municipalities of Dovre and Lesja, Oppland County, Central Norway. The area is surrounded by nature-protected areas (dark green), and after restoration the majority of the military area is included in the expanded protected areas

In 1999, the Norwegian Parliament decided to phase out a military training area in Hjerkinn, in the Dovre Mountains of central Norway, and “reset the area for civilian use and to restore the ecosystem to its original state and for future nature protection” (White Paper 1998; our translation). Military activity at the site started in 1923 and was expanded with heavy infrastructure from the 1960s. The site was intensively used until 2008 (Norwegian Defence Estate Agency 2020). The area comprises 165 km^2^ of alpine landscapes, including alpine heath, shrubland, peatland/wetland and barren land (Fig. 1). These mountain areas have been used for hunting and husbandry for centuries prior to the military use. Due its high natural and cultural values, the military area was surrounded by protected areas, into which the restoration would be incorporated.

The Hjerkinn project gives us a case study with which we can illustrate the use of boundary objects in restoration. A case study is an “empirical inquiry about a contemporary phenomenon (e.g., a ‘case’), set within its real-world context—especially when the boundaries between the phenomenon and context are not clearly evident” (Yin 2009, p. 18). The criterion for identifying a critical case is that it permits logical deduction, such as if an observation is or is not valid for the particular case, then it also applies—or does not apply to all other cases (Flyvbjerg 2006). As the largest restoration project in Norway, Hjerkinn involved contact with different actors and stakeholders with different backgrounds, organizational cultures, and perceptions of nature, which we believe makes the project a valid critical case for our study.

**Fig. 2:**
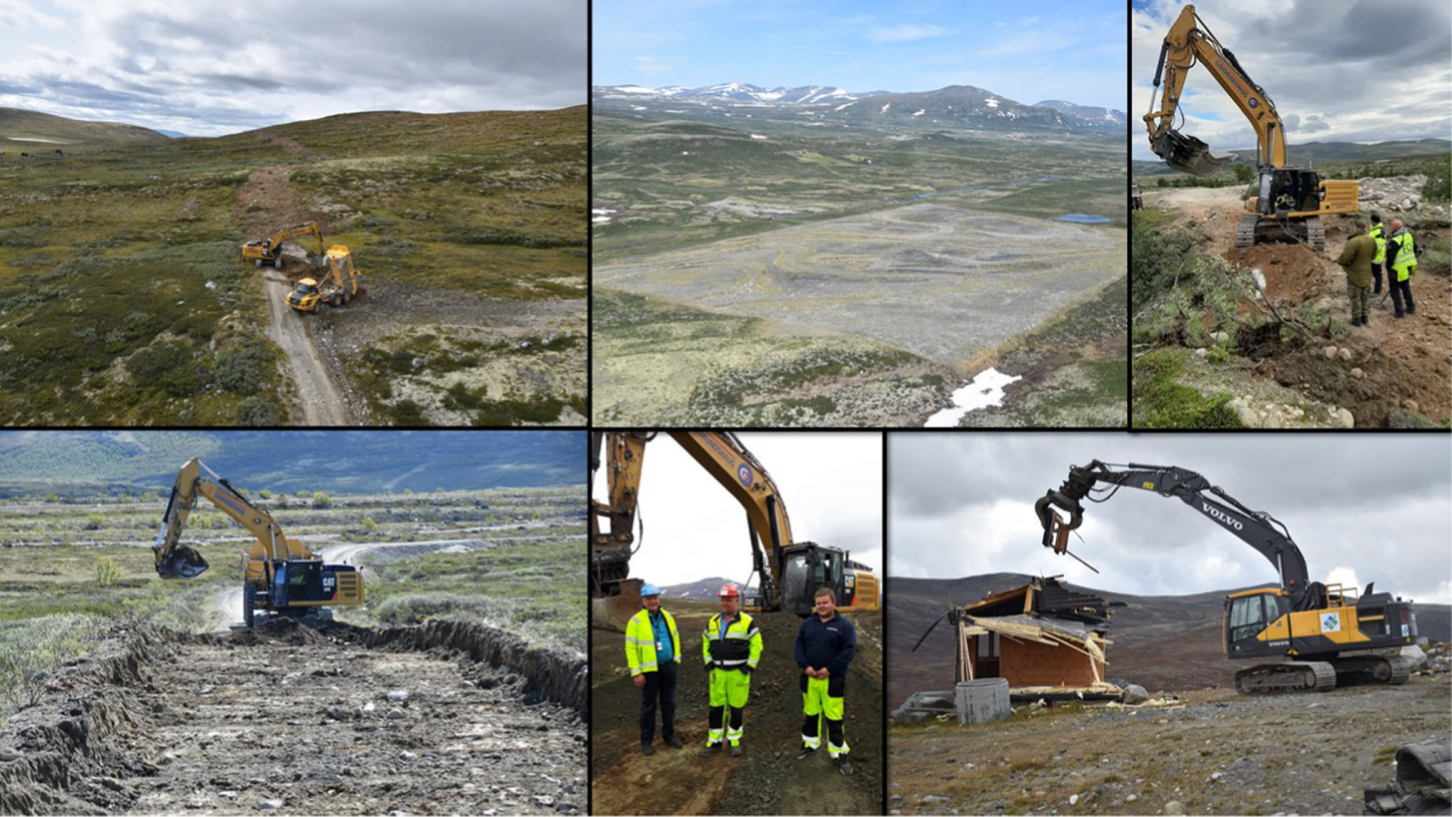
A core part of the restoration in Hjerkinn military training area is removing of roads and heavy infrastructure. Excavators and dumpers were used to reconstruct natural terrain (upper left) and remove added gravel (bottom left). The project also included restoration of large installations, such as the ammunition test field (upper centre) and pulling down more than 100 buildings (bottom right). Dialog between project owner, machine drivers, explosive experts, and restoration ecologist was crucial during the implementation stage (upper right, bottom centre). All photos credited: Dagmar Hagen

The Norwegian Defence Estate Agency has been responsible for the restoration project. The planning phase (1999–2007) included an environmental impact assessment (EIA), economic, strategic, and security planning, and the initiation of an environmental monitoring programme (Norwegian Defence Estate Agency 2020). The implementation period (2008–2020) included three subprojects; (1) the removal of undetonated explosives and fouled matter; (2) removal of pollutants and prevention of pollution; (3) removal of buildings, roads, borrow pits, and other installations, and the restoration of landscape, vegetation and ecosystem processes (Fig. 2, Martinsen and Hagen 2010; Hagen and Evju 2013; Norwegian Defence Estate Agency 2020). Concurrent with the restoration project, the local municipalities developed a land-use plan that focused on local economic development under future management (Dovre Municipality 2012). The county governor of Oppland created a plan for environmental protection of the area, in accordance with the Parliament’s decision. Accordingly, a diverse group of local, regional, and national actors had interests in the transformation of the military training area into a national park. In a formal legal procedure, the Norwegian Government established a national park and a special landscape protection area in large parts of the previous military area in April 2018 (Royal Decree 2018).

### Data collection and analysis

A document analysis was performed to map individual actors and information associated with the restoration project, including policy documents, plans, reports and assessments, as well as scientific papers. Semi-structured interviews were conducted with strategically selected informants from key actor groups, such as individuals with formal roles in the project (the Norwegian Defence Estate Agency and construction companies) and others (regional and local authorities, mountain boards (“Fjellstyre” in Norwegian), tourism industry), for a total of 14 informants (Table 1). Interviews were conducted using a five-section interview guide: (1) presentation of the purpose of the interview, (2) background information of the informants, (3) the informant’s views on nature, (4) the informant’s views on and knowledge of ecosystem restoration, and (5) the informant’s role and participation in the restoration at Hjerkinn. The personal interviews were audiotaped and transcribed. In addition, the authors’ personal experience from working directly within the project (D.H.), and local and regional newspapers, provided supplementary information on the process and local perceptions. Data from the interviews were organized as a combination of an “analysis continuum” to identify and add up categories and patterns of information (Krueger and Casey 2000) and “coding” of information into associations to help structure the empirical data (Tjora 2017). The patterns and structures of data were interpreted according to key concepts and viewpoints and sorted on the basis of their content and interrelationships (Kvale 1988, 1997). Observations and documents supported the categorization of the interviews.

**Table 1:** Informants interviewed in the Hjerkinn restoration project

Actor	Response method	Duration of interviews	N	Group
The Norwegian Defence Estates Agency (NDEA)	Personal interview	13–45 min	3	A
Statskog SF (the Norwegian state-owned land and forest company)	E-mail	Unknown	1	B
Oppland County Municipality	E-mail	Unknown	1	B
The County Governor of Oppland	E-mail	Unknown	1	B
Dovre municipality	Personal interview	30–50 min	2	B
Local stakeholder/farmer/etc	Personal interview	30–50 min	1	C
Lesja Mountain Board	E-mail	Unknown	1	C
Tourism industry enterprises	Personal interview	30–55 min	3	C
Construction company	E-mail	Unknown	1	A
Total number of informants			14	

Each informant was assigned to one of the following groups related to the project: *A *working on the restoration project (employed by The Norwegian Defence Estates Agency or consultants), *B *public agencies or municipalities, *C * tourism industry or other private enterprises

### Methodological considerations

We aimed for a diverse group of informants to gain broad insights into the views on the restoration process and determined the sample size as the point at which one extra informant would not change the overall picture (Kvale and Brinkmann 2009). The number of relevant informants in a single case study is limited, and we believe the interdisciplinary research team and composition of informants provided a robust design for the purpose of our study. Qualitative data pose some challenges with regard to conducting objective analyses, in part because the selection and wording of questions could have influenced the informants’ answers. Our interpretation of the answers also has a normative component. We acknowledge these possible pitfalls.

## Results and discussion

The Hjerkinn restoration included project preparation (based on the Parliament decision in 1999, until 2003), a project planning stage, including an environmental impact assessment, technical and economic planning, and dialog between stakeholders (2003–2008), and the actual intervention stage on the ground during 2008–2020. The main role for the Norwegian Defence Estates Agency was to operate and run the project. Stakeholders associated with the nearby communities were more focused on influencing the kind of values and end states that should be given priority during the restoration process. The construction companies and machine drivers were mainly focused on completing the practical interventions as described in the bid description. The restoration ecologists approached the project with the goal of obtaining measurable ecological outputs, including preparing for this outcome during the planning and implementation stages. All of the groups were committed to a common outcome, as formulated by the Parliament’s decision. Despite the span in approaches, backgrounds and motivations all the different actors and stakeholders expressed that they had to cooperate and support the project. Given the groups obligation for a common understanding we find it interesting to explore how these actors interacted in the complex Hjerkinn project. This combination of features makes the Hjerkinn project an interesting case to explore the idea of boundary objects in restoration ecology.

### Perceptions on nature

Some may perceive the act of restoration as bringing nature back to a kind of original state of wilderness, while others think of restoration as creating managed nature (Hagen et al. 2002). This was one of the major differences in the perceptions observed in our study, and can be linked to the different perceptions of nature overall (Cronon 1996; Castree 2001) among those involved in the Hjerkinn project. The “end state” of the project was to restore the area to a “wilderness” condition, which adds importance to how the different actors perceived nature. Based on our data this perception can be described along three interrelated dimensions:

- Untouched nature versus nature changed by humans
- Humans, as part of versus external to nature
- Use of nature versus conservation.

Some informants said they believed that humans are part of nature, while others said that nature should be “without physical encroachments and with absence of noise”, meaning free from any trace of human activities, as expressed by one nature manager from the county governor’s office in Oppland. This illustrates the conflicting opinions among informants. However, all informants said they believed the connection between nature and recreation were important to humans. Some informants, especially those representing local and regional stakeholders, perceived a cultural landscape as ‘nature’, and within this cultural understanding, domestic grazing is accepted as a part of ‘untouched nature.’ A related element concerned the question of how much the project should aim for recreating wilderness. Is true nature associated with wilderness without humans? The distinction as to whether or not humans could be considered a part of nature varied among the involved actors and stakeholders. To some degree this variation followed the view of untouched nature.

Another related dimension is the perception of use versus conservation of nature. In this area, traditional uses include outdoor recreation and hunting, tourism and domestic grazing. One representative from a nearby local community said: “The technical infrastructure is in my opinion perhaps something contrary to the traditional use. The nature on Dovrefjell is not wilderness. It has been used my humans in one way or another during all times” (our translation). The use-oriented perspective is rooted in local and regional stakeholders who were not active partners in the project, but who still tried to influence it. These groups were part of the political decision-making system, and were at the same time active users of the area being restored. However, these actors did not represent a unified perspective, but rather a diverse group of interests, with some mostly interested in hunting or grazing, while others interested in traditional outdoor recreation or commercial tourism with its need for associated infrastructure. This is an example of the variety of interests among the more use-oriented stakeholders.

Our data show that the informants had diverse perceptions of nature and different preferences regarding the level of interventions needed to achieve the restoration goal. The informants working inside the project had a higher degree of loyalty to the formal decisions, with less space for personal preferences. Informants “outside” of the project expressed the desire, when possible, to try to push the project in a direction that would reflect their preferences. Achieving project goals will therefore be influenced by people’s perception of the natural world.

In a complex, long-term project, differences in opinion will persist, even with good, strong participatory processes. The theoretical debate over planning and management generally holds that targeting consensus among participants will not provide a quick fix (Fyvbjerg 1998; Mouffe 1999; Flyvbjerg and Richardson 2002; Bäcklund and Mäntysalo 2010). Not accepting differences in how a project is perceived, despite comprehensive participation, may actually be seen as an exercise of power, or what Cooke and Kothari (2001) describe in their title “Participation—The new tyranny”. However, this also illustrates the possibilities offered by the “boundary object” concept, because it focuses on coordination and communication in situations where differences exist. In our view, the idea of restoring nature at Hjerkinn seems to be a core element in the boundary object of “ecosystem restoration”, while we have identified differences in the perception of “nature” in this specific case.

### Perception on goals and future use

The overall goal of the Hjerkinn project has been to restore the area for future nature protection and civilian use (White Paper 1998). All informants were positive towards the restoration process and its overarching goal. Their responses were somewhat more diverse when it came to specific project actions and the outcomes of the restoration, which to a certain degree coincided with their different views of nature.

One of the project’s largest, most demanding tasks was to reduce or eliminate the danger to humans and animals in the area by clearing away unexploded ordnance (explosives). The ordnance dated from the time when the military used the area a firing range. All of the study informants agreed on the importance of this safety issue. By the end of the project, a total of 19 000 unexploded munitions had been destroyed and 540 tonnes of metal had been removed from the area (Norwegian Defence Estate Agency 2020). Another main task was to restore the area to improve its natural qualities, a goal which all groups generally agreed upon. A nature manager from the county governor’s office said that restoration should “… return ecosystems, following disturbance, back to the most original state possible”. However, there were differences in views as to which areas and to what degree these restoration measures should apply. Some actors and stakeholders, especially local and regional representatives, wanted the area to be able to support future outdoor recreation, hunting and grazing by leaving existing infrastructure, which they maintained was compatible with nature protection and the establishment of conservation areas. These views were generally expressed by people who had historically used the area since before the military took over the land. They argued that the land should be restored primarily for traditional uses. At the same time, they said there was no need to return the landscape to its pre-firing-range condition, because they also wanted to retain roads that had been constructed by the military.

The main conflict in the restoration process centred around the degree to which the military’s technical infrastructure and roads should be removed. As one local informant said: “So the roads are the most important issue of conflict, I think” (our translation). Several local stakeholders wanted to keep the military roads, while the original project plan called for removing all technical infrastructure (White Paper 1998–1999). The arguments for keeping the roads were to create and develop tourism, give landowners access to grazing grounds, and give local people and visitors easier access to the area. The debate over roads intensified during the course of the project and eventually the original restoration plan was modified by the Norwegian Parliament, so that some roads will not be removed (White Paper 2018).

The informants also diverged regarding how strong they thought the restoration interventions needed to be to achieve the project goal. Some argued for just leaving the site and relying on nature to do the work (passive restoration) others supported more active restoration activities (Prach et al. 2020). As the project proceeded, the impact from active interventions became visual (Fig. 2) and got attention in local media, which might have reduced the diverging views. By the end of the project period, 5200 ha of land had been actively restored, and vegetation recovery had been well documented (Mehlhoop et al. 2018; Hagen et al. 2019).

There seemed to be a strong core of agreement on the main goals for restoration, but there were also conflicting issues related to final outcome and process. If we look at the restoration project as a boundary object, we can conclude that it was perceived from different angles, which might partly explain the divergent attitudes.

### Meaning and consequences of disturbed land

Most of the statements on how the informants perceived disturbed land related to the level of human influence and the human–nature dichotomy. In general, people’s perceptions of disturbed land were linked to physical evidence of human activities and landscape change.

The Dovre Mountains and Hjerkinn have a long history of human use, as evidenced by wild reindeer hunting structures that are thousands of years old, the more recent history of hunting and grazing, the military history, and today’s protection by law. However, the line between what is considered a valuable cultural landscape and what is considered a degraded landscape is not obvious (e.g. World Resources Institute 2021). There is a clear consensus among the informants concerning the high value of old and protected hunting installations. In Norway, the actively managed landscape, which includes grazing animals, is perceived as valuable (Daugstad et al. 2006; Handberg et al. 2018), and the majority of our informants considered domestic grazing to be a desirable activity in future landscapes at Hjerkinn. In contrast, most informants saw military installations, with the exception of roads, as not of value, and something that should be removed as part of the restoration. The decision to remove all evidence of the military installation at Hjerkinn was based on the predominant values and perceptions at that time (1999). Most actors saw the remnants from military use as destroying nature. One of our informants (a nature manager) set this in perspective: “The military installations can be preserved in photos from Hjerkinn” (our translation).

Military roads created the strongest controversy regarding what to remove and what to keep. The positive perception of roads by some of the informants was mainly based on the perceived need for future use rather than cultural heritage. This links perception of military roads to different attitudes towards people’s role in nature, the importance of wilderness as well as acceptance of human use.

We can observe a bounding core regarding giving value to old remnants from traditional use, as well as the acceptance of removing all military installations (except roads). There were diverging views regarding the need to remove military roads, and some informants perceived them as acceptable even in a natural area such as Hjerkinn.

### Boundary objects and restoration in the Hjerkinn military training area

Boundary objects bridge different perceptions by connecting different actors as well as the process around the object, such as the Hjerkinn project. In doing so, the object coordinates discourses and actions between actors with different values and goals. We observed both common understandings of the restoration process and conflicting issues. A boundary object functions as something that binds the different involved actors together. In this project, the actors may be grouped into those who were formally involved in the process, such as the Defence Agency, contractors and restoration scientists, and those more indirectly involved, such as local farmers, the tourism sector and the local community in general. The informants from nature and cultural heritage management were in the middle, because they may have legal authority to directly interfere in the process, but at the same time they are on the administrative fringe of the restoration project.

**Fig. 3:**
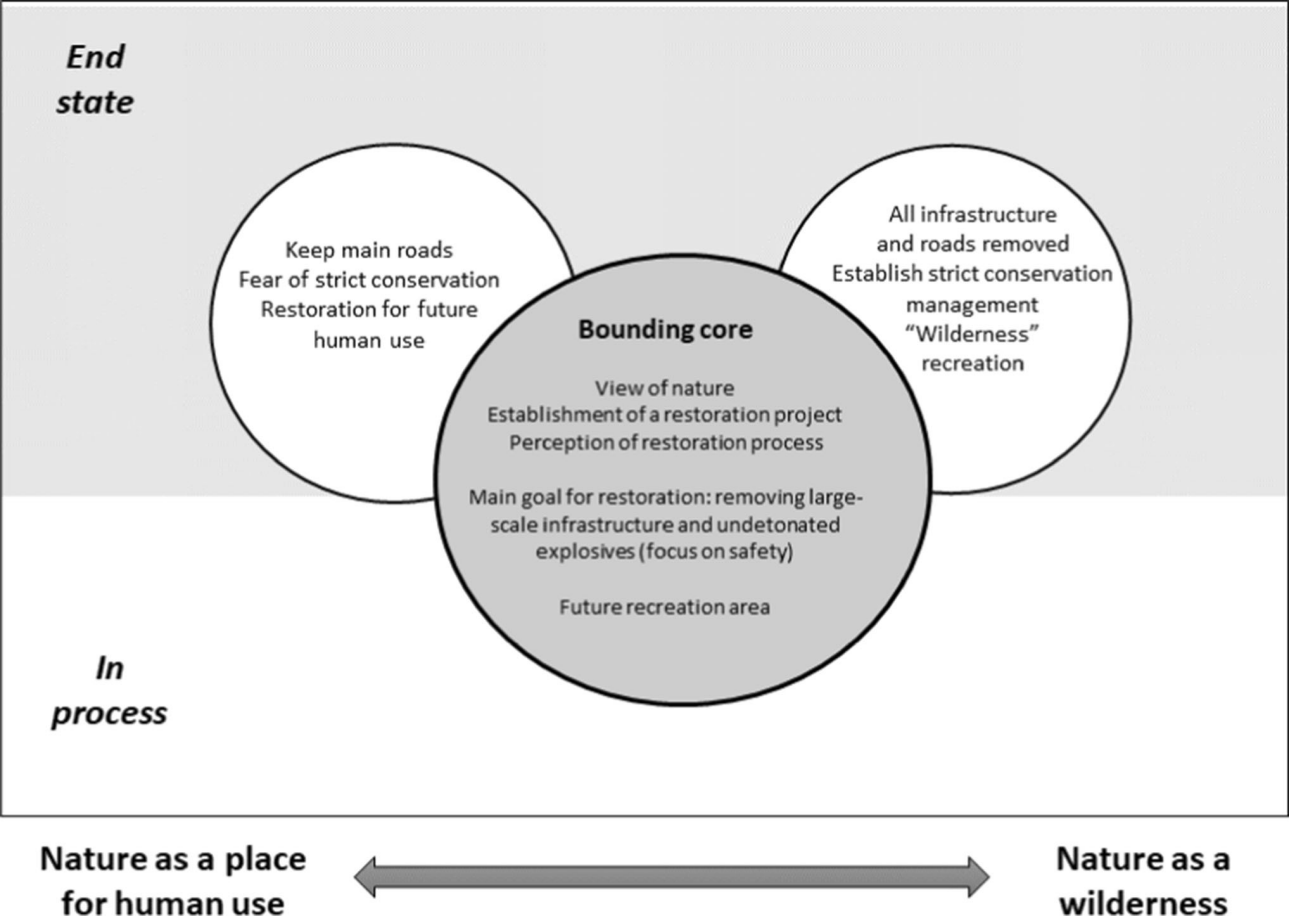
The Hjerkinn restoration project as a boundary object; its morphology with a common core and diverging elements

Ecosystem restoration as a boundary object has both a conceptual core, and related physical and organizational aspects. The conceptual core in this study is the restoration of nature in the case area, while the elements of deviation from the core were directed towards future use of the area (Fig. 3). Local stakeholders had a strong focus on future use and were negative towards “strict” conservation and wanted to keep the main roads in the area. Another group of stakeholders wanted to give priority to untouched nature and wanted to restore the area to wilderness, including removing all roads and only allowing wilderness recreation and traditional grazing in the area (Fig. 3).

The perceptions surrounding the Hjerkinn project had both a common core and elements that varied between actors, while the formal organization of the project interaction followed strict lines of command and responsibility. Our informants were partly formally employed or hired “inside” the project, and they partly belonged to groups “outside” the formal project (Table 1). Loyalty to political decisions seemed essential for the “insiders” in the project organization to feel a stronger commitment to making the restoration project a success. The more peripheral actors seem more motivated to influence the project in a desired direction. However, there was a consensus among all actors and stakeholder to implement the process, and nobody actually wanted the project to fail. This project has been a “flagship” for nature restoration in Norway, especially given the positive attention in media and from politicians and management authorities. In a project like this, the formal organization is important in the decision making surrounding specific actions. Loyalty to political decisions is essential for the “insiders”, as the success of the project would mean that they did a good job. However, at the same they must be responsive towards others (the “outsiders”) to get the project to run smoothly and reduce the risk of conflicts.

Based on our study, we believe that boundary objects can be a useful tool to identify the common denominator within a diverse group of actors and stakeholders with different interests, which can then be developed into a unifying commitment, like a ‘centripetal force’. If the stakeholder’s commitment is strong, the willingness to overcome conflicts and controversies may be greater in the presence of such a common commitment (Hartshorne 1950). The boundary object approach could be used as an analytical tool to investigate whether such a common “object” exists, from which coordination and communication can be developed, including both common and diverging elements. We believe that without such a common coordinating “object”, the risk for open conflicts may increase. In all management cases, antagonism will exist as long as there are actors with different interests, but by acknowledging a common mission, any future conflicts could be manageable. The existence of a boundary object will not guarantee that its coordinating and communicative power is strong enough to avoid conflicts. Yet, as an analytical concept, a boundary object can be used to analyse management processes, and perhaps also to design them in a way to actually establish functioning boundary objects.

We believe that restoration deals with desired states of linking restoration to preferences in the social system (cf. Hagen et al. 2002). The quality of the restoration depends on the identification of and movement towards such a desired state. Nurturing a common understanding would be useful to reduce conflicts, and would include communication on the content of the restoration project, including identification of the desired state towards which the restoration should work based on the relationship between a core and status of the diverging dimensions (Fig. 3). Hence, improving the quality of a restoration project would also involve reaching a common understanding of a desired state.

The analytical use of the concept of boundary object can give a better understanding of restoration processes, and the improved design of these processes can be used as a tool both to reduce conflict and to enhance the quality of restoration projects. The boundary object is a tool that targets the process, but it does not state what the end restoration stage (or level) should be. However, no process will be better than the willingness of participants and actors to work towards formulating a common desired state.

## Conclusion

This study used the concept of boundary objects to analyse ecosystem restoration in a specific case at Hjerkinn, in the Dovre Mountains in Norway. *First*, we identified a clear core perception: that all informants accepted and supported the establishment of the restoration project, and supported many of the activities to remove large installations, limit pollution, and remove undetonated explosives. However, there were differences of opinion regarding the end result of the restoration, along the following dimensions:

- Internal actors versus external stakeholders
- Project activities versus future use

*Second*, we believe the concept of boundary objects is a useful analytical tool. The ideas behind the restoration of the area formed the basis for the project and directed the actors and stakeholders’ behaviour. The “commonness” of a boundary object can be an important property for restoration projects. However, if controversies are too strong, it would be difficult to establish a coordinating and communicative “boundary object”, and the coherence of a project could disappear. In this regard, a “boundary object” appears to be a promising analytical concept for studying the implementation of restoration projects. *Finally*, we believe that the analytical use of the concept of boundary object could be a useful tool to determine when restoration projects are being run well and when they are failing. No processes will be better than the willingness of the involved participants and actors. The existence of a common project will always have the possibility of disintegrating if a common core is not established and nurtured.
